# iPSC-Derived Hepatocytes as a Platform for Disease Modeling and Drug Discovery

**DOI:** 10.3389/fmed.2019.00265

**Published:** 2019-11-15

**Authors:** James L. Corbett, Stephen A. Duncan

**Affiliations:** Department of Regenerative Medicine and Cell Biology, Medical University of South Carolina, Charleston, SC, United States

**Keywords:** iPSC, hepatocyte, differentiation, CRISPR-Cas9, disease modeling, drug screens

## Abstract

The liver is one of the largest organs in the body and is responsible for a diverse repertoire of metabolic processes. Such processes include the secretion of serum proteins, carbohydrate and lipid metabolism, bile acid and urea synthesis, detoxification of drugs and metabolic waste products, and vitamin and carbohydrate storage. Currently, liver disease is one of the most prevalent causes of mortality in the USA with congenital liver defects contributing to a significant proportion of these deaths. Historically the study of liver disease has been hampered by a shortage of organ donors, the subsequent scarcity of healthy tissue, and the failure of animal models to fully recapitulate human liver function. *In vitro* culture of hepatocytes has also proven difficult because primary hepatocytes rapidly de-differentiate in culture. Recent advances in stem cell technology have facilitated the generation of induced pluripotent stem cells (iPSCs) from various somatic cell types from patients. Such cells can be differentiated to a liver cell fate, essentially providing a limitless supply of cells with hepatocyte characteristics that can mimic the pathophysiology of liver disease. Furthermore, development of the CRISPR-Cas9 system, as well as advancement of miniaturized differentiation platforms has facilitated the development of high throughput models for the investigation of hepatocyte differentiation and drug discovery. In this review, we will explore the latest advances in iPSC-based disease modeling and drug screening platforms and examine how this technology is being used to identify new pharmacological interventions, and to advance our understanding of liver development and mechanisms of disease. We will cover how iPSC technology is being used to develop predictive models for rare diseases and how information gained from large *in vitro* screening experiments can be used to directly inform clinical investigation.

## Introduction

The liver is an endoderm-derived organ with dual endocrine and exocrine roles that are vital for the maintenance of physiological homeostasis. It is the largest organ in the body and is responsible for a several metabolic processes including, carbohydrate and lipid metabolism, bile acid and urea synthesis, detoxification of drugs and metabolic waste products, and vitamin and carbohydrate storage (1). The main parenchymal cells of the liver are hepatocytes which make up approximately 80% of the total volume with the remaining 20% made up of non-parenchymal cells including biliary epithelial cells, sinusoidal endothelial cells, hepatic stellate cells and Kupffer cells (2). Because of its vital regulatory function, congenital or drug induced perturbations of the liver are often fatal or require lifelong management with liver transplantation being the only treatment for end-stage liver failure. Currently 4.5 million people in the USA live with chronic liver disease and it is the 9th leading cause of death overall (3).

One of the biggest bottlenecks in identifying novel therapeutics for the treatment of liver disease is our inability to accurately model the physiological functions of the liver *in vitro*. Many cell culture models rely on immortalized or cancer cell lines which, while ideal for early stage proof of concept experiments, do not express metabolic enzymes at levels resembling primary hepatocytes, with the exception of the HepRG cell line which expresses high levels of the fetal enzyme CYP3A7 but low levels of the adult enzymes CYP1A2 and CYP2D6 (4, 5). Because of their tumorigenic origin, cancer cell lines also show signs of immaturity and have dysfunctional apoptotic pathways making them resistant to toxicological insult and unsuitable for many drug screening applications (5–8). Additionally, because cancer cell lines are often derived from single hepatomas, they show limited genotypic variability and are not representative of broad patient populations. Subsequently many drugs pass through the earlier stages of a clinical trial only to be found ineffective or toxic at later stages. It is estimated that 40% of new chemical entities that undergo preclinical safety studies in animals and 89% of new chemical entities that enter clinical trials will fail due to unforeseen toxicity (9, 10).

Primary liver cells taken from human cadavers or medical biopsies are currently the gold standard for investigations of human disease and development of novel pharmacological treatments, as they are morphologically and biochemically similar to healthy human cells *in-vivo* (11). The relatively robust CYP450 enzyme activity in primary hepatocytes makes them the most viable candidates for *in-vitro* hepatotoxicity studies but their propensity to de-differentiate and rapidly lose mature function, as well as the difficulty in acquiring a readily available supply of cells makes them unsuitable for many other research applications (12–14). Recent advances allowing prolonged expansion of previously cryopreserved primary human hepatocytes by dedifferentiation to a proliferative state followed by redifferentiation, may help researchers attain large numbers of primary hepatocyte like cells (15). However, fresh primary liver cells are still difficult to acquire (especially from patients with particularly rare disease), display functional variability between donors, and don't maintain their phenotype in culture for more than a few days, making them unsuitable for a number of disease studies or drug screens (16, 17).

One potential solution to the lack of available healthy primary human liver cells is to differentiate pluripotent stem cells toward a hepatic fate. Historically stem cells derived from the inner cell mass of the embryonic blastocyst were utilized in differentiation protocols, but since the development of protocols to reprogram somatic cells to pluripotent stem cells, the use of induced pluripotent stem cells (iPSCs) has become more common (18). One of the main advantages of iPSCs, which we will cover later in this review, is the potential to derive them from adult human tissue, including those harvested from patients with rare genetic disorders. iPCSs can also proliferate indefinitely and with careful handling maintain relatively stable genomic transcriptional and epigenetic profiles, making them an ideal source of cells for disease modeling and large scale screening experiments (19).

## Hepatic Differentiation of iPSCs *in vitro*

Current *in vitro* hepatic differentiation protocols aim to mirror the *in vivo* developmental processes that form the liver during embryogenesis. Most of our understanding of hepatogenesis has been elucidated from work using non-human developmental models including mouse, rat, chick, zebrafish, and xenopus laevis (20–23). The majority of differentiation protocols use a combination of growth factors and small molecules to recapitulate the various hepatic developmental stages: from pluripotent embryonic cells in the blastula, to definitive endoderm, early hepatic progenitors, bipotent hepatoblasts, immature hepatocytes, and finally mature hepatocytes (24–27). Hepatic induction protocols often use Activin A, FGF2 and BMP4 to induce definitive endoderm differentiation, FGF2 and BMP4 for hepatoblast differentiation and HGF and OSM for hepatic maturation (26). These protocols produce induced hepatocytes with many of the characteristics of hepatocytes isolated from human livers, including lipid storage, albumin secretion, accumulation of glycogen, active uptake of low-density lipoproteins, and synthesis of urea (26, 28).

Despite success in generating cells that retain many characteristics of hepatocytes, most iPSC to hepatocyte differentiation protocols produce cells that more closely resemble fetal or newborn hepatocytes (29, 30). For example, the physiologically relevant expression of phase I and phase II metabolic enzymes, which are responsible for a majority of the steps involved in drug metabolism, is enhanced in iPSC derived hepatocytes over most hepatoma cell lines but is comparatively reduced compared to primary hepatocytes (4, 17). Such reduced expression argues that iPSC derived hepatocytes are not yet an optimal platform for all aspects of preclinical pharmaceutical development (31). Nevertheless, recent efforts to improve culture conditions have yielded advances, indicating that iPSC-derived hepatocytes have considerable potential to predict drug toxicity in humans and could improve the efficiency of drug discovery (32–34).

A common criticism of many hepatic differentiation protocols is that the cells are often plated on a 2D plastic cell culture surface that is usually pre-treated with tumor derived extracellular matrix (ECM) to aid in cell attachment and survival. Current 2D culture models arguably don't support the complex interplay of cell-cell and cell-ECM interactions vital for cell polarization and maturation of iPSC derived hepatocytes (35). Analysis of primary human hepatocytes cultured on tumor derived ECM indicates that de-differentiation occurs at a greater rate when compared to primary liver derived ECM (36). Notably, mechanical properties of cell culture plastic are also dissimilar to the normal liver microenvironment, being much stiffer and less elastic than conditions *in vivo* which may impact the differentiation state of cultured cells (37). Recently, differentiation protocols have begun to take into account the developing liver's physiological microenvironment and have adapted accordingly. Novel cell culture platforms are being developed that utilize ECM components such as collagen, laminin, and fibronectin to mimic the liver microenvironment which are also incorporated into 3D scaffolds that allow cells to arrange themselves in a more physiologically relevant manner (38–40).

New cell culture platforms are increasingly being constructed from decellularized ECM scaffolds derived from human cadavers, mouse, rat, and pig livers which are then directly seeded with iPSCs or deconstructed and used to pre-treat specific culture platforms (41–43).

Synthetic 3D scaffolds are also being developed that permit more physiologically relevant arrangements and interaction of cultured tissue. Cell culture compatible hydrogels allow for cells to be suspended in a liquid gel which later solidify, allowing for cells to aggregate and proliferate into the hydrogel matrix (44). Cells can also be seeded directly onto pre-constructed polymer scaffolds which are either entirely synthetic, or are hybrid structures treated with ECM components prior to cell seeding (45, 46). Hepatocytes differentiated in 3D ECM based scaffolds showed significantly greater expression of mature liver markers and greater CYP450 enzyme activity compared to standard 2D cultures (44, 47–49). While these newer differentiation formats show promise there remains a difficulty in reliably sourcing cadaveric material, inter-batch reproducibility, and scalability in most platforms. Accessing ECM derived from humans can be particularly challenging and prevents widescale adoption in standardized differentiation protocols.

Another related approach to enhancing the hepatic differentiation of cells *in vitro* is to culture iPSCs in platforms that allow for spontaneous 3D aggregation and differentiation. Cellular aggregations, known as organoids, can self-organize into multicellular structures containing functional tissues that resemble those in the adult organ (50). Hepatic organoids derived from iPSCs have been developed by combining hepatic progenitor cells with human umbilical vein endothelial cells and human mesenchymal stem cells (51). After 48 h of co-culture the cells aggregated and formed 3D structures with primitive endothelial networks and the gene expression profile showed similarity to fetuses between 22 and 40 weeks. Organoids transplanted into mice connected rapidly with the host vasculature and showed enhanced maturity over aggregates co-cultured without endothelial cells. A recent study by Pettinato et al. (52) demonstrated that *in vitro* culture of iPSC derived hepatocytes with human adipose microvascular endothelial cells (HAMECs) resulted in spontaneous generation of CD31 positive sinusoid like structures as well as greater and more persistent upregulation of functions associated with mature liver cells. Co-cultures of iPSCs and HAMECs demonstrated enhanced expression of coagulation pathway components, expression of factors involved in thrombolysis and haemostasis, albumin, and urea production, increased CPY450 activity, and glycogen and lipid storage. Many functions associated with hepatic maturity in the co-cultured organoids were equivalent or enhanced over those in 2D cultures of both adult and fetal primary human hepatocytes. Finally, when organoids were transplanted into damaged rat livers, the organoids were able to significantly improve survival over a 14 day period, indicative of physiologically relevant liver function (52). Hepatic organoids derived from iPSCs show advanced hepatic differentiation when compared to their 2D counterparts and have the advantage of being scalable for clinical and high throughput applications (53). As iPSC differentiation protocols improve to the point of resembling primary tissue, careful control over culture conditions will have to be exercised in order for gene expression and CYP450 activity to resemble normal liver function. With advances in hepatic differentiation conditions, 3D culture platforms and generation of multi-tissue organoids it seems likely that improved iPSC–derived hepatocyte function will soon resemble that of primary human hepatocytes, facilitating their use in drug discovery, disease modeling, and regenerative therapy.

## Modeling Disease Using Patient Derived iPSC Differentiated to a Hepatic Lineage

As mentioned previously, somatic cells can be harvested from specific individuals and transformed into induced pluripotent stem cells. This approach was first described by Takahashi et al. (18) via transduction of four transcription factors: Oct3/4, Sox2, Klf4, and c-Myc, and also by Yu et al. (54) using Oct4, Sox2, Nanog, and Lin28. The reprogrammed cells were morphologically and functionally similar to embryonic stem cells and could differentiate into all three germ layers. Since the discovery of these initial factors, non-integrative approaches for cellular reprogramming have been developed (55) and iPSCs have been reprogrammed from cells that don't require invasive harvesting such as keratinocytes, peripheral blood cells and renal epithelial cells from urine (56–58). The relative ease with which iPSCs can now be derived and the flexibility to readily differentiate iPSCs into numerous tissue types (providing a robust differentiation protocol is available) makes them favorable for use in a wide range of drug discovery and disease modeling applications. There has also been recent success in reprogramming adult somatic cells into hepatocytes using lentiviral transduction of select hepatic transcription factors, resulting in cells which perform similarly to primary human hepatocytes (59). Although hepatocytes derived from iPSCs don't yet display a fully mature phenotype, it is important to recognize that they outperform many other liver cell models and the extent of differentiation achieved using current protocols is sufficient to model most elements of liver disease. One of the most compelling reasons to use iPSCs is the ability to source them from patients with rare diseases. Patient derived cells can be reprogrammed to iPSCs, expanded, and differentiated, thereby providing a limitless supply of disease specific hepatocytes. Here, we will review a number of diseases which have been modeled using iPSC hepatocyte–like cells derived from patients. Table 1 lists the growing number of liver diseases that can now be modeled *in vitro* using iPSC derived hepatocytes.

**Table 1:** Liver disease and potential therapeutic interventions modeled using patient derived iPSCs.

**Disease**	**Gene/Toxin**	**Intervention**	**References**
Alpha-1-antitrypsin deficiency	*AAT (p.E342K) and (p.E342K)*	Five drugs identified including Carbamazepine: Increased autophagy mediated degradation of folded AAT proteins.	(60–62)
Alpha-1-antitrypsin deficiency	*AAT (p. E342K)*	Gene correction of AAT mutant iPSCs via homologous recombination of the wild type sequence using Zinc Finger Nuclease and a piggybac transposon prevented aggregation of mutant polymeric AAT in hepatocytes.	(63)
Alpers-huttenlocher syndrome	*POLG (p.A467T)*	Cyclosporine A: rescued Valproic acid induced apoptotic sensitivity in Alpers-Huttenlocher hepatocytes.	(64)
Citrin deficiency	*SLC25A13*	Treatment of cells with the PPAR-α agonist Wy-14591resulted in partial reduction in cellular triglyceride accumulation	(65)
Citrullinemia type 1	*ASS1 (p.G259X/R304W)*	L-arginine: Reduced ammonia and rescued ureagenesis in Citrullinemia type 1 hepatocytes.	(66)
Crigler najjar syndrome	*UGT1A1 (p.L413P)*	None	(60, 67)
Familial hypercholesterolemia	*LDLR*	Cardiac glycosides: Increase proteolytic turnover of ApoB.	(60, 68–70)
Glycogen storage disease type Ia	*G6PC*	None	(60)
Glycogen storage type Ib	*SLC37A1 (c.1124-2A > G)*	None	(67, 71)
Hemophilia A	*F8*	None	(72)
Hemophilia B	*F9 (c.278-3 > G)*	None	(73)
Hepatitis C	*HCV*	INF-α and ribavirin resulted in decreased HCV RNA in both the cellular fraction and the culture medium	(74)
Hyperargininemia	*ARG1 (p.A298P/p.R21X)*	None	(75)
Idiosyncratic drug-induced hepatotoxicity	Pazopanib	None	(76)
MELAS syndrome	*m.3398T > C*	AMPK inhibition enhanced glycogen storage and lactic acid turnover.	(77)
Malaria	*P. falciparum*	Atovaquone and primaquine treatment resulted in the reduction in size and distribution of *P. falciparum* exoerythrocytic forms in treated cells.	(78)
mtDNA depletion syndrome 3 (MTDPS3)	*DGUOK (p.W166X;H167LfsTer213)*	NAD Increased ATP Production and rescues mitochondrial function in engineered mutant DGUOK iPSC derived hepatocytes and DGUOK deficient rats.	(79)
Niemann–pick disease type C	*NPC1 (p.I1061T)*	Combinatorial treatment with HP-β-cyclodextrin and carbamazepine: Restored cholesterol and autophagy defects in Niemann–Pick disease type C.	(80)
Niemann-pick disease type C	*NPC1 (p.S667L/C1161Y)*	2-HB-γ-cyclodextrin: Reduced cholesterol accumulation more effectively than HP-β-cyclodextrin and restored the functional and molecular abnormalities in the Niemann–Pick disease type C hepatocytes.	(81)
Paracetamol/acetominophen induced liver toxicity	Paracetamol/acetominophen	Anti-microRNA-324: Increased expression of the Phase II enzyme SULT2A1 enhancing cell survival.	(82)
Pompe disease	*GAA (p.R854X)*	None	(83)
Primary hyperoxaluria type 1	*AGXT (p.I244T)*	Lentiviral delivery of wild-type *AGXT* cDNA driven by a transthyretin promoter.	(84)
Progressive familial intrahepatic cholestasis type 2	*ABCB11 (c.24 C>A/c.2417 G>A)*	4-phenylbutyrate: Rescued membrane bile salt export pump expression level and biliary excretion capacity.	(85)
Tyrosinemia Type 1	*FAH (p.Q64H)*	None	(60, 67)
Tangier Disease	*ABCA1 (p.E1005X* and *p.S2046R/p.K531N)*	None	(86)
Wilson's disease	*ATP7B (p. R778L)*	Curcumin: Enhanced copper export from Wilson's disease hepatocytes.	(87, 88)
Wolman disease	Free fatty acid exposure	FGF19 treatment suppresses lipid accumulation and reduced liver organoid stiffening in the disease model.	(89)
Zellweger spectrum disorder	*PEX1* c.2097_2098insT p.I700fs and c.2916delA p.G973fs	None	(90)

Multiple research groups have generated iPSCs from Familial Hypercholesterolemia (FH) patients (60, 68, 69). FH is caused by mutations in the low-density lipoprotein receptor (*LDLR*) and is characterized by elevated serum LDL-cholesterol resulting in severe cardiovascular disease. Cayo et al. (68) derived iPSCs from the fibroblasts of “JD” a 14 year old male patient with FH and differentiated them toward a hepatic fate. The differentiated iPSC hepatocytes showed the comparable hepatic profile to non-disease control iPSCs and displayed a striking eight-fold increase in the level of secreted ApoB-100, indicative of the FH disease phenotype. JD FH hepatocytes failed to uptake fluorescently-labeled LDL–cholesterol particles and when treated with lovastatin, a drug which in healthy cells upregulates *LDLR* mRNA expression and subsequently increases LDL–cholesterol uptake, the JD FH cells increased *LDLR* mRNA expression but failed to increase the uptake of LDL-cholesterol. These results indicated that the patient derived iPSC hepatocytes recapitulated key elements of the FH pathophysiology *in vitro*. Similar results were obtained by other researchers using iPSC hepatocytes derived from independent patients (60, 69).

Alpha-1-Antitrypsin (AAT) deficiency is a congenital liver disease which commonly arises from a G > A point mutation at codon 342 (p.E342K) that promotes spontaneous polymerization and intracellular aggregation of AAT. The buildup of polymerized proteins leads to cellular overload, hepatic cell death, fibrosis and liver failure (63). Researchers were able to differentiate AAT deficient iPSCs to hepatocytes which showed comparable hepatic differentiation, but had a significant accumulation of AAT specifically in the patient derived cells (60, 63). Using a Zinc Finger nuclease, Yusa et al. (63) were able to correct the mutant p.E342K *AAT* gene and restore the structure and function of the AAT protein preventing accumulation in the modified hepatocytes and eliminating the disease phenotype. Tafaleng et al. (91) also carried out an investigation to model personalized variations in AAT deficiency using iPSC lines derived from patients with variable presentation of disease severity. In AAT deficiency the distinction between severe and mild presentations of the disease is relatively binary, with mild forms of the disease displaying little AAT accumulation of the misfolded proteins and greater accumulation in the severe form. As AAT deficiency is relatively common in persons of European descent, the authors had ready access to multiple lines derived from several genetically unrelated patients. They found that iPSCs derived from patients with mild forms of AAT deficiency with no outward disease symptoms were able to degrade and secrete the immature/misfolded proteins relatively efficiently, whereas in severe mutations the AAT was degraded slowly and its immature form was localized to pre-Golgi compartments, resulting in aggregation and aberration of cellular function and morphology (91). The patient derived iPSC–derived hepatocytes in this study recapitulated the range in clinical severity remarkably well *in vitro*. Such studies demonstrate the utility of iPSCs in the prediction of disease susceptibility and outcome. In the future it is likely that iPSCs will be useful to predict the degree of clinical intervention necessary for patients, based on genetic screening of various disease severities and the severity of iPSC–derived hepatocytes.

Historically, hepatoma cell lines have been used for *in vitro* cell culture models to study infectious disease, but these cells do not always accurately recapitulate the functioning of primary human hepatocytes making the study of hepatotropic infections such as Hepatitis C (HCV), which has a narrow host range, challenging (92). However, researchers found that hepatocytes derived from iPSCs provided an available source of cells with a consistent genetic background that were permissible to HCV infection and completion of the HCV life cycle (93, 94). Furthermore, treatment of HCV infected iPSC derived hepatocytes with the anti HCV agents INF-α and ribavirin resulted in decreased HCV RNA in both the cellular fraction and the culture medium, validating iPSC derived hepatocytes as a model for studying HCV infection and treatment (74). Malaria research has also been hampered by poor availability of donor primary human hepatocytes. Researchers found that iPSC derived liver cells were permissible for infection by the human malarial parasites *P. falciparum* and *P. vivax* and supported maturation, especially at later stages of hepatic maturity (78). However, infection with parasites in the iPSC derived hepatocytes showed limited ability to respond to the established anti-malarial drug primaquine, which the researchers attributed to lack of activation by drug metabolizing enzyme activity. The parasite drug response was enhanced by a small molecule driven maturation of the iPSC-hepatocytes, but still remained incomplete compared to primary cells (78). These results provide encouraging evidence that iPSC derived hepatocytes can be used to investigate therapies for malarial infection, but more robust differentiation protocols may need to be developed before they can replace primary hepatocytes as the gold standard.

Researchers are also beginning to use iPSCs reprogrammed from large patient populations along with hepatic differentiation protocols to elucidate the genetic underpinnings of complex diseases. Pashos et al. (95) generated iPSCs from peripheral blood mononuclear cells isolated from 91 individuals from predominantly African American and European American donors. They performed genome-wide eQTL and ASE mapping to associate specific novel allelic variants [single nucleotide polymorphisms previously identified by genome-wide association study (GWAS)] with variations in gene function associated with lipid metabolism. They confirmed these functional associations by engineering specific allelic variations into mice using CRISPR-Cas9 and observed the same variations in lipid profiles predicted from the human iPSC screen. These results confirm the utility of iPSC derived liver cells for use in investigating the genetic underpinnings of complex phenotypes which have previously been too obscure to unravel.

## High Throughput Screening Using iPSC Derived Hepatocytes

Most current iPSC to hepatocyte differentiation protocols are readily scalable, meaning that they can be used to generate hepatocytes in a range of miniaturized tissue culture formats including 96-well and 384-well formats (24, 96). Miniaturized tissue culture formats are readily convertible for use in high throughput platforms to assess drug toxicity as part of a pre-clinical investigation or to identify novel compounds for the treatment of disease from pre-existing drug libraries. High throughput screens allow rapid assessment of a large number of compounds for their effect on *in vitro* disease phenotype. For high throughput screening to be a viable approach to drug discovery, investigators need (1) a scalable model to which the compounds of interest are applied such as cells, media, or protein isolates, (2) a robust and unambiguous endpoint indicating the state of the biological process of interest, and (3) a cheap, rapid, and scalable assay to detect the desired endpoint. Complex assays or platforms do not lend themselves to the screening of large numbers of compounds.

Small molecule screens have been used in the past to identify whether hepatic culture platforms accurately model drug toxicity *in vitro* (33, 97, 98) as well as identify molecular pathways that enhance or inhibit hepatocyte differentiation (96, 99, 100). Screens using specific classes of compounds have also been used to identify optimum efficacy from families of drugs (80, 81) as well as identify novel small molecules for the treatment of congenital liver disease (61, 70, 79) (Table 2). iPSC–derived hepatocytes incorporated as part of a platform for drug discovery show great potential for the identification of completely novel therapies for previously untreatable disease.

**Table 2:** High throughput platforms for drug discovery and toxicology assessment using iPSC derived hepatocytes.

**Purpose of screen**	**Assay type**	**High throughput procedure**	**Outcome**	**Reference**
Identification of drugs for treatment of Alpha-1-Antitrypsin (AAT) deficiency	Small Molecule Screen	AAT Patient iPSC- derived hepatocytes were treated with a drug library containing 3,131 clinical compounds and analyzed with immunofluorescence against cellular AAT. High Throughput microscopy was used to identify small molecules that reversed AAT accumulation.	5 drugs identified that consistently reduced AAT accumulation. One of the drugs identified was Carbamazepine which has previously been used to clear protein aggregates *in vivo* thus validating the screens application for drug discovery.	(61)
Prediction of Drug Induced Liver Injury using embryonic stem cell derived hepatocytes	Toxicity Screen	Twenty known hepatotoxins were screened using primary and stem cell-derived hepatocytes for 1, 4, or 7 days at concentration of 0.1, 1, 10, 25, 50, 100, or 200 μM. Cellular ATP was used to determine whether the drugs at each time point reduced cells to at least 50% viability (IC50).	At day 1 fewer compounds were toxic to stem cell derived hepatocytes (45%) compared to primary hepatocytes (60%), after 4 days of treatment both platforms showed similar sensitivity showing 65 and 60% sensitivity, respectively. After 7 days both platforms equally successfully identified 75% of the compounds as toxic.	(97)
Small molecule screen for hepatocyte proliferation and maturation.	Small Molecule Screen	12,480 small molecules were screened for their ability to enhance the expansion or function of cultured primary hepatocytes and then applied to iPSCs during hepatic differentiation.	2 small molecules assigned FH1 and FPH1 were identified that enhanced the maturation of cultured iPSC derived hepatocytes	(100)
iPSC-Human Hepatocyte-based micropatterned co-cultures platform for high throughput toxicity screening	Toxicity Screen	iPSC derived hepatocyte like cells were co-cultured with 3T3 fibroblasts and exposed to 47 compounds, 37 known to be toxic, 10 known non-toxic.	iPSC and primary human hepatocyte based co-culture platforms had sensitivities of 65 and 70%, respectively, for the 37 known hepatotoxic compounds tested. Neither model showed a false positive to the non-toxic compounds.	(33)
High-throughput confocal microscopy analysis of toxic compounds on the morphology and viability of iPSC derived 3D liver spheroids	Toxicity Screen	Representative set of 48 compounds 42 known to be cytotoxic or hepatotoxic and 6 with no known toxicity were analyzed for their effect on Human iPSC-derived hepatocyte spheroids.	36 of the toxic compounds resulted in toxicity effects in the spheroid assay (86%) with no false positives from the non-toxic compounds. 21 compounds showed a trend toward stronger toxicity effects in 3D culture when compared to 2D cultured cells. iPSC derived spheroids showed a markedly less toxic response to most anti-proliferative agents compared to HepG2 spheroids.	(98)
Identification of drugs for the treatment of Familial Hypercholesterolemia	Small Molecule Screen	2,320 small molecules were screened to identify compounds that could reduce the levels of apoB in hepatocytes derived from Familial Hypercholesterolemia iPSCs.	13 small molecules were identified which reliably reduced apoB. Five of the identified compounds were cardiac glycosides which reduced apoB via enhanced proteolytic turnover.	(70)
Small molecule screen to elucidate unknown cellular mechanisms that underly liver development	Small Molecule Screen	1,120 small molecules with well-defined molecular pathways were screened against iPSC during hepatic differentiation to determine which mechanisms were required for the maintenance of HNF4α	132 small molecules were identified that impacted HNF4α expression. Gene ontology analyses linking interactions between small molecules and proteins revealed heat shock protein 90 alpha family class B member 1 (HSP90β) played a role in HNF4α regulation. Disruption of HSP90β led to a reduction in HNF4α and co-immunoprecipitation indicated HSP90β plays a role in regulatingHNF4α protein folding.	(96)
Genetic and chemical high throughput screen to identify reagents that enhanced hepatic differentiation	Genetic and small molecule screen	A Genome wide CRISPR-Cas9-lentiviral screen along with an iPSC Albumin reporter line was used in a high throughput format to identify *HDAC3* as a regulator of hepatic differentiation.	A high throughput small molecule screen found that treatment of iPSCs with the HDAC inhibitor CI-994 resulted in greater expression of several hepatic markers, as well as reduced expression of *AFP*, compared with control cells.	(99)
Small molecule screen to identify compounds that could reverse the disease phenotype of Mitochondrial DNA Depletion Syndrome (MTDPS3)	Small Molecule Screen	2,400 drugs, a majority of which had been approved for use in humans were screened against hepatocytes derived from iPSCs with a CRISPR loss of function mutation in DGUOK.	15 drugs were identified which enhanced endogenous ATP production in the diseased cells. The candidate drug NAD was selected for further assessment and successfully reversed the disease phenotype in DGUOK mutant rats	(79)

Choi et al. (61) used iPSC derived hepatocytes from patients with AAT deficiency to analyze accumulation of the protein aggregates in a high throughput *in vitro* model of the disease. Cells were treated with a drug library containing 3,131 clinical compounds and analyzed with immunofluorescence microscopy to identify small molecules that could reverse AAT accumulation. In total, 262 compounds were identified that reversed AAT accumulation by at least 50%. They went on to repeat the screen with 43 compounds that were not reported to induce major side effects and tested the drugs on four different AAT deficient patient iPSC lines. After repeat screening, five drugs were verified to consistently reduce AAT accumulation. One of the drugs was Carbamazepine which had already been reported to clear protein aggregates in non-human models of the disease, thus validating a high throughput screening approach in identifying novel, clinically useful drugs to clear AAT accumulation.

Cayo et al. (70) developed a screen using iPSC-derived hepatocytes from a patient with FH to screen a library of 2,320 small molecules that reduced the levels of apoB, which is the central protein component of (v)LDL, in the media of cultured FH iPSC-derived hepatocytes. Of the drugs that were screened, 13 reproducibly reduced apoB production. Most of the identified drugs were cardiac glycosides which lowered apoB by enhancing its proteolytic turnover. Cardiac glycosides are used to treat heart failure, which allowed the results to be validated in humans by retrospective analyses of clinical records. Individuals treated with cardiac glycosides for heart failure also showed a significant reduction in serum LDL-C, indicating that cardiac glycosides could potentially be used for treatment of FH (70).

**Figure 1:**
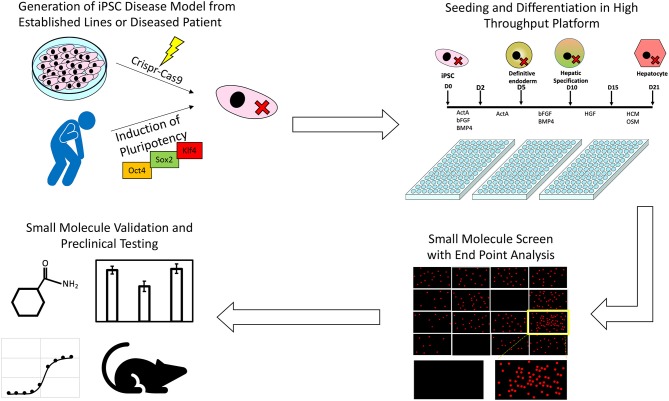
An illustration of the process through which iPSCs along with high throughput screening platforms can be used to identify novel treatments for inborn errors in hepatic metabolism and other liver diseases.

The process by which iPSCs along with high throughput screening platforms can be used to identify novel treatments from pre-existing drug libraries is summarized in Figure 1. To date only a handful of potential liver disease treatments have been investigated using a high throughput screening approach, but as more robust, scalable hepatic differentiation protocols are developed along with more scalable endpoint assays, the number of investigations successfully identifying novel treatment modalities is sure to increase.

## Models for Investigating and Treating Rare Liver Disease Using CRISPR-Cas9 Engineered iPSCs

Recent advances in CRISPR-Cas9 based gene editing has allowed for cheap, efficient, highly targeted manipulation of the human genome and offers the potential to genetically modify a target cell line as needed. CRISPR-Cas9 works by cutting double stranded DNA at precise locations which stimulates cellular repair mechanisms, either by non-homologous end joining, which often creates disruptive insertion or deletion mutations, or homology-directed repair (HDR), which can be exploited to insert specific sequences into target genes (101). CRISPR-Cas9 can be used to manufacture specific disease-causing mutations with variable severity using a single cell line. This approach removes uncertainty arising from differentiation potential between mutant cell lines, providing a standardized genomic background against which the mutant phenotype can be compared. Omer et al. (102) used CRISPR-Cas9 with HDR to correct a three base pair homozygous deletion in the *LDLR* gene in iPSCs derived from familial hypercholesterolemia patients. When differentiated to hepatocytes the corrected cells displayed mature low-density LDLR protein induction in response to lovastatin and restored LDLR mediated endocytosis of LDL. Omer et al. (102) demonstrated the principle that CRISPR-Cas9 can be used to rescue the function of hepatocytes derived from diseased iPSCs and compared the rescue effect within the same genetic background. It is not unreasonable then that CRISPR-Cas9 technology could also be used to engineer known genetic diseases in established cell lines and compare the phenotypic variations to cells within the same genetic background.

Previously, one of the greatest challenges in the study and treatment of rare disease was reliable access to tissue samples from diseased patients, often the low frequency of occurrence combined with sporadic access to, and fragility of, diseased patients meant that primary cells were impossible to acquire. The 2002 Rare Disease Act defines a rare disease as any that affect a population of <200,000 individuals in the USA. Many rare diseases are known as “Orphan Diseases” as few resources are available to develop therapies for such a limited number of patients. Academic institutions however are not restricted by profit margins and with recent advances in using iPSCs in drug screening platforms, are well-placed to develop novel treatments for orphan diseases. One such orphan disease is mitochondrial DNA depletion syndrome type 3 (MTDPS3) which arises from mutations within the deoxyguanosine kinase (*DGUOK)* gene. *DGUOK* encodes the mitochondrial deoxyguanosine kinase, an enzyme that phosphorylates purine deoxyribonucleosides that are essential for mtDNA replication and repair (103, 104). A majority of MTDPS3 patients suffer from a broad spectrum of clinical phenotypes, the severity of which appears to relate to specific allelic variants within the gene. In most cases, patients with a *DGUOK* mutation develop liver failure, but more severe mutants often lead to multisystem failure with a correspondingly poorer prognosis (105). There is currently no cure for MTDPS3 and available treatments rely on symptom management or liver transplantation. To address this Jing and colleagues developed CRISPR-Cas9 engineered loss-of-function mutations of DGUOK in iPSCs, which were then differentiated into hepatocytes. The DGUOK mutant iPSCs displayed many characteristics of the MTDPS3 disease phenotype *in vitro* including mtDNA depletion, reduced expression of mitochondria encoded electron transport chain proteins, reduced ATP output, increased reactive oxygen species accumulation, and increased extracellular lactate accumulation. A high throughput drug screen was carried out with 2,400 drugs from the Spectrum collection, over half of which have been approved for human use in the United States (The Spectrum Collection–Microsource Discovery Systems Inc.^1^). Cellular ATP production was measured to determine if any drugs reversed the MTDPS3 phenotype. The researchers identified 15 drugs which enhanced ATP by at least 20% over vehicle treated cells. The identified drugs included nicotinamide adenine dinucleotide (NAD), which increased the expression of all mitochondrial encoded electron transport chain genes examined. NAD is the bioactive form of Niacin, an FDA approved drug with minimal toxicity, which is important if NAD is to be used by patients on a long term basis. Treatment of DGUOK knockout rats with the NAD precursor Nicotinamide Riboside also resulted in increased ATP production and significantly improved electron transport chain complex activity (79). These results confirm the utility of combining CRISPR-Cas9 generated iPSC disease models and drug screens to identify novel treatments for rare diseases. Such studies pave the way for further investigation of the mechanisms, mutation dependent severity, and treatments of rare diseases using CRISPR-Cas9 engineered mutant iPSCs.

## Conclusion

Patient derived iPSCs have become a vital tool for researchers trying to uncover the regulatory mechanisms behind hepatic development, the underpinnings of congenital disease, and the mechanisms of drug metabolism and toxicity. iPSC derived hepatocytes provide a stable, readily available cell source for applications previously requiring human primary hepatocyte or hepatoma cell lines, including the investigation of defects in lipid metabolism (70, 106), protein accumulation (61), mitochondrial defects (77, 79), and toxicity screening (33, 97, 98). It is worth noting that no differentiation protocol yet produces hepatocytes that are functionally identical to primary human liver cells, particularly in regard to CYP450 enzyme expression which is essential for drug metabolism (107). However, newer differentiation protocols using 3D co-culture of iPSC hepatocytes with cells from multiple lineages show promise in enhancing hepatocyte metabolic function that more closely resembles the physiology of hepatocytes in the liver (52). Development of a scalable iPSC hepatic differentiation protocol that recapitulates physiological CYP450 enzyme activity would open the door for reliable, high throughput, toxicity testing of candidate drugs in pre-clinical studies, potentially saving billions of dollars in pharmaceutical development costs. Additionally, more robust differentiation protocols would allow for assessment of subtle variations in drug metabolism due to pharmacogenetic polymorphisms and variable disease phenotypes arising from allelic variations (108). To date, most investigations of disease causing mutations have focused on single allelic variations in coding regions. Recent advances in next generation sequencing approaches combined with improvements in the reliability of CRISPR-Cas9 gene editing, and the derivation of iPSC disease hepatocytes have been used to identify complex non-coding gene regulatory elements that result in disease. A recent study was able to validate iPSC derived hepatocytes as a platform to identify non-coding SNPs from GWAS resulting in lipid metabolic defects (95). Combinations of GWAS with high throughput screens for novel therapeutics could be used to investigate and tailor treatments for patient populations with rare or idiosyncratic disease presentations. Furthermore, specialized drug repurposing libraries consisting of compounds already approved for clinical use by the FDA could be prioritized for screening, providing drugs for rapid acceleration to clinical application without the need for slow and costly preclinical safety assessments (61, 79). As advances are made in robust derivation of iPSCs from primary cells, *in vitro* hepatocyte differentiation, CRISPR-Cas9 targeting efficiency, and scalability of disease phenotype detection protocols, high throughput drug screening will become an increasingly viable option for the study and treatment of a multitude of diseases.

## Author Contributions

JC wrote first draft of manuscript, edited, designed figures, and tables. SD edited the manuscript, contributed sections, and reviewed figures and tables.

### Conflict of Interest

The authors declare that the research was conducted in the absence of any commercial or financial relationships that could be construed as a potential conflict of interest.

## Footnotes

^1^The Spectrum Collection - Microsource Discovery Systems Inc. Available online at: http://www.msdiscovery.com/spectrum.html (accessed October 10, 2019).
